# Weston-Super-Mare

**Published:** 1889-09

**Authors:** Charles Vernon Hitchins

**Affiliations:** Medical Officer of Health for Weston-super-Mare


### WESTON-SUPER-MARE.

BY

CHARLES VERNON HITCHINS,
*Medical Officer of Health for Weston-super-Mare.*

BUT few people can now say, as they did some few years ago, "Where on earth is Weston-super-Mare?" for this bright little town has in a brief period grown from a village composed of a few fisher-huts, scattered here and there about its sandy wastes, to a fashionable seaside resort of no mean renown, bidding fair to beat in the struggle for popularity many, if not all, of its West of England rivals.

In the month of August of 1819, when the justly celebrated Mrs. Piozzi, Dr. Johnson's friend, was sent to Weston for her health by her Bath physician, she found the village very far indeed from the madding crowd; and in a letter to Sir James Fellows she pithily describes her temporary abode: "The breezes here are most salubrious; no land nearer than North America when we look down the Channel; and 'tis said that Sebastian Cabot used to stand where I now sit, and meditate his future discoveries of Newfoundland. Who would be living at Bath now? . . . . I enquired for books. 'There were but two in the town,' was the reply: a Bible and a *Paradise Lost.* They were the best, however. No market; but I didn't care about that."

In 1811 the census-returns showed a population of 163, and this number increased by leaps and bounds till in 1881 it reached 12,872; and it is now estimated, not by *rule of thumb*, but by those most likely to know the real facts, that the normal residential population is 18,000 at least, increased by several thousands during the summer season. The marked increase in size is clearly demonstrated by the figures of the assessable value published in the yearly synopsis of the town-accounts by the local authorities. Thus, in 1842, when the first Improvement Act was passed, the rateable value was £7,964, while in March, 1889, it was £82,802 2s. 3d.

Weston-super-Mare is situated on a spur of the Mendips, and is built partly on the southern slopes of the well-wooded Worlebury Hill and partly on the level immediately beneath, stretching year by year further along the coast-line of the Bristol Channel and also inland, and adding villa to villa and street to street with astonishing rapidity. The distance from London is 135 miles, and from Bristol only 18. The journey down from the former place is most easily accomplished. The intending visitor can leave the Great Western Railway Terminus at Paddington by the wonderful "Zulu"—that smooth-flying three o'clock afternoon express—and, after changing at Bristol, stand on the Weston platform at half-past six, with the salt breezes from the sea blowing in his face, and reach his hotel or his lodgings in time for his dinner or supper, whichever he may call his evening meal. From Manchester, Weston is also remarkably easy of access. The merchant can accomplish a morning's work, leave the city by the 12.10 train, and arrive at 6.35 at the West of England Brighton. The villa residences are for the most part built of the blue limestone procured from the town quarry, and have a solid and substantial appearance, the stucco or cemented front which frequently covers builders' shortcomings being very seldom seen. They dot the side of the hill in a most picturesque manner, and being sheltered from the north and east by the rising ground at the back, and embowered in trees and shrubs of luxuriant growth, afford shelter all the year round to the most delicate. Why go to the South of France and endure the discomforts of a foreign country and the doubtful drainage, and of dwelling amidst unsympathetic strangers, when the invalid can live at home amongst friends, with English comforts and a climate resembling the health-resorts of the so-called Sunny South, in the sheltered situations of Weston? The fact is, the home health-stations are far too little recognised and understood as winter-resorts.

The sea recedes a considerable way in Weston Bay, leaving exposed to the sun and the sea-breezes a large tract of mud, at which some laugh, some sneer. Let them. Unhealthy? No: this mud is one of Weston's most important features; for the Atlantic breezes blowing across it catch up a large amount of ozone and iodine, and then discharge their load into the lungs of visitors and residents alike, making the weak strong and the strong feel as giants, bringing back the glow of health to the jaded and overworked dwellers in cities, and making the convalescent forget that he has ever lain on the bed of sickness. Three hundred acres of well-planted woods, through which the public are permitted to roam at will, form no small attraction to this health-resort. Few seaside places on our coasts can boast of possessing such a pleasing and beautiful adjunct, and Weston can well look with pride and thankfulness upon her free wooded slopes; and those with health impaired, to whom the windy seaside is too invigorating, can revel in the sheltered moss-grown paths and shady avenues of these Weston woods without fear of being confronted by an indignant keeper, or being frightened with a "Trespassers will be prosecuted" board.

The first questions usually asked in these latter years of the nineteenth century, when everybody, more or less, is educated up to a high standard of hygienic knowledge, are: Is the town well drained? And is there a good water supply? To both questions we can safely answer in the affirmative. As to drainage, large sums of money, something like £50,000, have been expended in the construction and ventilation of the drains. The outfall is some two miles from the town, at the mouth of the Uphill River; and the sewage is discharged by means of a penstock into the ebbing tide only, when it is carried by the swift current well clear of Weston Bay and down the Bristol Channel or the Severn Sea, as it was formerly called. The remarkable rise and fall in the tides in this Channel are particularly favourable for the effectual removal of town-sewage; at the Weston pier-head, for instance, the tide rises on an average some 38 feet. About six years ago the open ventilation system was adopted, upon the recommendation of the Local Government Board in London, and it is found to answer satisfactorily and well. In the place of open gratings in the streets, which were considered objectionable by some, the local authorities have, after obtaining the consent of private owners, wherever opportunity occurred, erected large ventilating shafts, six or eight inches in diameter, against the sides of buildings in not too conspicuous places; and these carried well above the eaves and away from all windows have been found to answer every purpose, and to relieve the sewers of gas very effectually. There is also a system of flushing the main drains, which works to great advantage. An excellent arrangement is also maintained by which any house can be overhauled by the sanitary inspector of the Local Board, free of charge, at the request of the landlord or of an intending lessee or purchaser; and the inspector, if desired, and if the premises inspected be in a satisfactory sanitary condition, will give a certificate thereof. These certificates are yearly becoming more and more appreciated; for lodging-house and hotel keepers find it much to their advantage to have one framed and hanging in their hall for any lodger's inspection; while purchasers of houses buying subject to such a certificate may be sure that they will not be made liable for heavy drainage-expenses for years to come. The local authorities have formulated strict rules for the proper ventilation and disconnection of all pipes, it being their continual anxiety to maintain a high standard of healthiness. As to the water, which is, as it always should be, in the hands of the town, there is an unbounded supply, though the quality is somewhat hard. It bubbles up in two wells connected together and cut in the mountain-limestone, each well being only 25 feet deep. The quantity obtainable from this source is 75,000 gallons an hour—about three times the present requirements of the town. Within the last two years new mains have been laid throughout the town to meet the growing needs of the population, and larger and most powerful engines have been erected to pump the water into the two reservoirs, one of which is situated half way up the hill, the other upon the summit. The quantity of water is so copious that there has never been any occasion to place the inhabitants upon short commons even during the driest season; and the house-supply is constant night and day. The land, several acres in extent, immediately above and around the wells and pumping-station, has been recently acquired by the town-authorities, upon the advice of the Local Government Board, to prevent any possible risk of the source of supply being in any way contaminated by building-operations being carried on in too close proximity.

The value of a health-resort is more or less judged by its death-rate; and when this test is applied to Weston, it will be seen that the result is satisfactory. The death-rate calculated upon the present population shows for the past three years an average of 13·3; for 1888 it was only 12 per thousand; while for the quarter ending March last it was 9·6. The rate from zymotic diseases for the past three years averaged 1·3; from phthisis, ·98; and from respiratory diseases, 2·7. The corresponding rates for all England for 1886, the last published, were 2·6, 1·7, and 3·7.

The town is well provided with medical institutions, the most important being the General Hospital, which, commencing years ago in a very small way, now contains 31 beds, and receives patients from all quarters. It has an excellently-fitted children's ward; and in connection there is a provident dispensary, to which belong more than three thousand members. The Infectious Diseases Hospital is situated about a mile from the town, and is built according to the official requirements. It contains eight beds, is kept in complete order, and is open to all classes. On the beach midway between Weston and Uphill is situated a stately pile of buildings known as the West of England Sanatorium, one of the largest and best-appointed of the kind in England: more than a thousand patients pass through its doors every year, and these are convalescents only. It does a great work. Visitors are invited to see it in the afternoon of any week-day, and to attend the chapel-services on Sunday.

The climate may be described as equable, dry, and bracing. The mean annual temperature is almost identical with that of London; but as it is instructive to note how fallacious is this bare *datum*, I venture to quote from statistics kindly supplied by Mr. R. S. Culley. By comparing the mean monthly temperature of the two places it is shown that during the summer months—viz: May, June, July, August, and September—Weston is the cooler; whilst from October to March it is warmer than London, and in April the monthly means are equal; the maximum winter-difference being 8°, and the maximum summer-difference 12°, both in favour of Weston.

The character which Weston has for dryness depends not so much on the record of its rainfall as on the porous nature of its limestone soil and the evaporating power of its breezes. Visitors are often surprised to find that after heavy rain an hour or so suffices to render the roads attractive to the pedestrian or cyclist. The driest months are March, April, and June. Most rain falls in October. Of wind there is seldom or never any lack in Weston. Even on the hottest day a refreshing breeze is generally to be felt; and at intervals, especially about March and mid-October strong W. and S.W. gales visit us. These constant atmospheric movements, combined with and aided by the extensive rise and fall of the tide and the expanse of ozone-yielding surface thereby exposed, the clearness of atmosphere and brightness of sunshine, combine to produce a state of chemical activity to which I should attribute the bracing nature of the climate.

As regards the medical aspects of Weston-super-Mare, it follows from the preceding remarks that those diseased constitutions requiring a warm and equable, yet bracing and dry climate do best here.

Thus, strumous patients with enlarged glands or joints are frequently seen amongst our visitors and schools, and in the convalescent homes.

Patients with delicate chests derive much benefit from the warm and tonic climate, but do well to avoid the March winds; and consumptives in the later stages should not come here except in the full summer. I may notice in passing the great comparative rarity of phthisis amongst the residents, a fact which the statistics partly show, and one which is remarked on by medical men and others accustomed to hospital and private practice elsewhere.

A large section of our invalids come hither from Bath to recruit, after undergoing the anti-arthritic treatment of the justly-famed mineral springs there; and to these rheumatic and gouty patients a word of caution is necessary; viz., regarding the hardness of the water. If they consume this in large quantity, it should be decalcified by the simple process of boiling and subsequent filtration.*

There is an old precept, "Take a blue pill the first night you get to the seaside"; and although I do not counsel its literal enforcement, yet a judicious aperient during the first few days of a visit here would often obviate an attack of liver-derangement in those naturally predisposed thereto, and in others who acquire this

* A proposal for softening the water at the springs is under consideration.

predisposition by yielding, "not wisely but too well," to the appetising influences of the air. Another of the primary effects of coming here is a tendency in the majority of visitors to increased sleep: this tendency may last from two or three days to as many weeks, after which they usually become acclimatised. Most nervous affections (especially those of a neurasthenic type) do well here, e.g. persons suffering from over-brainwork, from neuralgia (not due to carious teeth and other local causes), cases of hysteria, hysterical aphonia, and such like. In this connection I may perhaps mention, as somewhat suggestive and curious, the fact that from time to time instances occur of parents coming here with families sufficiently old to make them consider a nursery in the new home altogether superfluous, yet, after a while, they discover that fresh parental joys await them.

Children seem to thrive here in a remarkable degree, whether more than at other seaside places I cannot say; but the healthy look of the natives and the benefit that the children of visiting families derive are matters of frequent comment.

There is no lack of amusement for those who cannot exist without bands and illuminations and excitement; and while some prefer the fashionable sea-front lounge or the streets of shops, others can inspect one of the most perfect of the ancient British dry-walled fortresses extant, with its pit-dwellings, which immediately overhangs the town, or seek out the quiet beauties of the surrounding country; but the health-giving breezes, the bright sunlight, the shady woods, and the crisp waves breaking on the sands and pebbly beaches are for all.

Note.—I am indebted for valuable assistance in the preparation of this paper to Mr. Ernest E. Baker, F.S.A., and Dr. Rossiter.

